# The acute pressure natriuresis response is suppressed by selective ET_A_ receptor blockade

**DOI:** 10.1042/CS20210937

**Published:** 2022-01-05

**Authors:** Geoffrey J. Culshaw, David Binnie, Neeraj Dhaun, Patrick W.F. Hadoke, Matthew A. Bailey, David J. Webb

**Affiliations:** University of Edinburgh/British Heart Foundation Centre for Cardiovascular Science, The Queen’s Medical Research Institute, The University of Edinburgh, 47 Little France Crescent, Edinburgh EH16 4TJ, U.K.

**Keywords:** blood pressure, endothelin receptor antagonists, hypertension, pressure natriuresis, sodium homeostasis

## Abstract

Hypertension is a major risk factor for cardiovascular disease. In a significant minority of people, it develops when salt intake is increased (salt-sensitivity). It is not clear whether this represents impaired vascular function or disruption to the relationship between blood pressure (BP) and renal salt-handling (pressure natriuresis, PN). Endothelin-1 (ET-1) regulates BP via ET_A_ and ET_B_ receptor subtypes. Blockade of ET_A_ receptors reduces BP but promotes sodium retention by an unknown mechanism. ET_B_ blockade increases both BP and sodium retention. We hypothesized that ET_A_ blockade promotes sodium and water retention by suppressing PN. We also investigated whether suppression of PN might reflect off-target ET_B_ blockade. Acute PN was induced by sequential arterial ligation in male Sprague Dawley rats. Intravenous atrasentan (ET_A_ antagonist, 5 mg/kg) halved the normal increase in medullary perfusion and reduced sodium and water excretion by >60%. This was not due to off-target ET_B_ blockade because intravenous A-192621 (ET_B_ antagonist, 10 mg/kg) increased natriuresis by 50% without modifying medullary perfusion. In a separate experiment in salt-loaded rats monitored by radiotelemetry, oral atrasentan reduced systolic and diastolic BP by ∼10 mmHg, but additional oral A-192621 reversed these effects. Endogenous ET_A_ stimulation has natriuretic effects mediated by renal vascular dilation while endogenous ET_B_ stimulation in the kidney has antinatriuretic effects via renal tubular mechanisms. Pharmacological manipulation of vascular function with ET antagonists modifies the BP set-point, but even highly selective ET_A_ antagonists attenuate PN, which may be associated with salt and water retention.

## Introduction

Hypertension is the single biggest contributor to global disease burden and remains a major risk factor for cardiovascular disease despite current multimodal therapeutic strategies [1,2]. Blood pressure (BP) rises with salt intake and this effect is exaggerated in a significant minority of people [3]. The mechanisms of such salt-sensitivity remain contentious. Since salt intake commonly far exceeds recommended upper limits [4], there is an unmet need to further understand and target the disruption to normal sodium handling that leads to salt-sensitivity.

The computational model of Guyton and others places the kidneys as the central regulator of long-term BP, achieved through relative homeostasis of extracellular fluid. Thus, the expansion of intravascular volume, after sodium and water ingestion for example, causes renal arterial pressure to rise, which in turn induces sodium excretion [5]. The pressure natriuresis (PN) relationship can be measured empirically and is attenuated in settings of experimental hypertension. The Guyton model has influenced hypertension research for more than 50 years but is challenged by an alternative hypothesis that salt-sensitivity is a manifestation of impaired vasodilatation rather than inadequate sodium and water excretion [6]. This is based on observations that dilatation of the systemic vasculature normally accommodates salt-induced intravascular volume expansion to buffer the effect on BP, while, in salt-sensitive individuals, vasodilatation is impaired and BP rises. The relative merits of these hypotheses are vigorously debated [6–8] but, in combination, they suggest that the ideal therapeutic agent for salt-sensitivity would be one that promotes dilatation, while at the same time promoting PN. In this respect, endothelin (ET) receptor antagonists offer considerable potential.

ET-1 promotes vasoconstriction via vascular ET_A_ receptors, vasodilatation via endothelial ET_B_ receptors, and natriuresis via ET_B_ receptors in the renal collecting duct [9–11]. The latter response is enhanced by a high salt diet [12]. This suggests that blockade of ET_A_ receptors, but not ET_B_ receptors, would promote vasodilatation and allow the systemic vasculature to accommodate increased blood volume following sodium ingestion, while not interfering with natriuresis that decreases blood volume. This is supported by clinical studies in which selective targeting of ET_A_ receptors reduced BP in patients with resistant hypertension [13–16], and, in patients with non-diabetic chronic kidney disease, restored nocturnal dipping in BP [17], a marker of daytime sodium excretion [18]. However, deployment of selective ET_A_ receptor antagonists has been stifled because ET_A_ antagonists also consistently promote sodium and water retention [19,20]. Therefore, we hypothesized that ET_A_ blockade promotes sodium and water retention by suppressing PN. We also explored whether suppression of PN might reflect off-target ET_B_ receptor blockade.

## Materials and methods

### Experimental animals

Experiments were performed in accordance with the UK’s Animals (Scientific Procedures) Act (ASPA) under a UK Home Office Project Licence. All protocols were reviewed by the University’s Animal Welfare and Ethics Review Board prior to experimentation (357-LF2-16 and 381-LF2-18).

Adult male Sprague Dawley rats, weighing 250–300 g, were purchased from Charles River UK, and transported to Edinburgh under conditions specified in the UK’s Animal Welfare Act, 2006. Rats were maintained on standard chow (0.25% sodium) and water *ad libitum* and were housed in rooms with a 12-h light cycle (lights 7 a.m. to 7 p.m.) at 21 ± 1°C and 50% humidity. In total, 59 rats were used in the present study.

Experiments and assays were performed with the operator blind to treatment group, and assays were performed in duplicate.

### Renal function studies

The original protocol for PN [21] was adapted, as described previously [22]. It was applied to adult rats under non-recovery anaesthesia induced by intraperitoneal thiopental; (50 mg/kg, 50 mg/ml; Archimedes Pharma, Reading, U.K.) and maintained by intravenous thiopental of the same concentration. Rats received an intravenous (jugular) infusion of physiological saline (pH 7.4; 1 ml/hour/100 gbw) containing 2% (weight:volume) bovine serum albumin to maintain euvolaemia. 0.25% fluorescein isothiocyanate (FITC)-inulin (Sigma-Aldrich Company Ltd, Gillingham, U.K.) was added to the infusate to measure glomerular filtration rate (GFR) by inulin clearance. An arterial line (carotid) was used for blood sampling and real-time BP measurement via a calibrated transducer and multi-channel data acquisition system (Powerlab; ADInstruments, Oxford, U.K.).

Following laparotomy, ligatures were pre-placed around the coeliac and mesenteric arteries, and the aorta distal to the left kidney. Left renal artery blood flow (ml/min) was measured by a calibrated Doppler ultrasound probe (PR-probe; Transonic, Ithaca, U.S.A.) positioned around the renal artery stripped of periarterial fat and nerves [21]. Laser Doppler spectroscopy measured regional changes in renal blood flow [22,23]. A laser Doppler patch probe (MSP100XP; ADInstruments) was glued to the dorsal surface of the left kidney to measure cortical flux, and medullary flux was measured by a needle probe (MNP110XP; ADInstruments) inserted through the capsule to a depth of ∼5 mm and orientated toward the hilus. Urine was collected from the left ureter.

Following surgery, all rats received slow (over 5 min) intravenous injection of one of four treatment options: atrasentan (endothelin A (ET_A_) receptor antagonist, (AbbVie Ltd., Maidenhead, U.K.) 5 mg/kg dissolved in 1 ml of vehicle [24]), A-192621 (ET_B_ receptor antagonist, (AbbVie Ltd.) 10 mg/kg dissolved in 1 ml of vehicle [24]), combined atrasentan and A-192621, or vehicle (1 ml of physiological saline, pH 7.4 and 50 µl of 70% ethanol).

After 30 min for equilibration, urine collections were made over three sequential periods, each lasting 30 min. After the first (baseline) collection, BP was acutely increased by ligation of the cranial mesenteric and coeliac arteries (ligation 1), and urine was collected. The distal aorta was then ligated (ligation 2), after which the final urine collection was made. The entire procedure was performed under homeostatic temperature control at 37°C.

Urinary sodium concentrations were measured by a sodium-selective electrode (9180 Electrolyte Analyser; Roche Diagnostics Ltd., Burgess Hill, U.K.). Mean renal blood flow values were calculated, along with urine flow rate (UV), urinary sodium excretion rate (UNaV) and fractional excretion of sodium (FENa). GFR was calculated from the fluorescent signal at wavelength 538 nm following excitation at 485 nm (Infinite M1000 Pro; Tecan Group Ltd., Männedorf, Switzerland)

### Continuous measurement of BP in conscious rats

Radiotelemetry devices (TA11-CA P40; Data Sciences International, Hertogenbosch, Netherlands) were pre-calibrated and implanted into the abdominal aorta of male Sprague Dawley rats under anaesthesia with inhalational isoflurane (Isoflo; Zoetis Animal Health Ltd., Sandwich, U.K.) using aseptic technique [22]. Recovery from surgery was aided by warm airflow and analgesia with 0.5mg/kg s.c. buprenorphine (Buprecare; Animal Care UK, York, U.K.) every 12 h for 3 days.

The start of every day was defined as the start of the dark period at local time 7 p.m. Baseline systolic BP (SBP), diastolic BP (DBP) and heart rate data prior to salt supplementation have been published [22] and are not presented here. Radiotelemetry recording was then extended in the same rats for three consecutive weeks to limit the number of animals used, and we present these data. During the first of these weeks, rats received additional dietary salt after the 6 p.m. but before the 7 p.m. data collection, in order to determine the BP response to salt-loading. The salt was in the form of an ice-cube (∼10 ml)-sized gelatin (Dr. Oetker UK Ltd, Leyland, U.K.) block containing soluble meat extract (80 mg sodium chloride/ml; 410 mg beef extract/ml; Bovril; Unilever UK Ltd, Leatherhead, U.K.) so that, for every rat, daily sodium intake was 140 mg of sodium/day based on 15 g/day daily intake of pelleted food (Benevenga et al., 1995). This equated to a 1% sodium diet, four times the concentration of sodium in the unsupplemented diet. One gelatin block was placed in every rat’s cage on top of a cardboard tube used for environmental enrichment. Voluntary, rapid consumption of the block was visualized in every case.

To determine whether the BP response to salt-loading was modified by ET receptor antagonists, the gelatin block was supplemented [25] with atrasentan (5 mg/kg) during the second week, and then, during the third week, atrasentan and A-192621 (10 mg/kg). Data presented here were acquired during the middle five days of each week (1 kHz over a 1-min period in every hour). Data from the first and seventh days were excluded to remove confounding stress effects associated with weekly staff changes and routine husbandry.

At the end of the experiment, rats underwent euthanasia by carbon dioxide asphyxiation followed by cervical dislocation.

### Statistics

Data are expressed as mean ± standard deviation and analysed with Minitab 17 (Minitab Ltd, Coventry, U.K.) and GraphPad Prism 8.4 (GraphPad Software, La Jolla, U.S.A.).

Numbers of rats varied between groups according to experimental losses, and control groups contained larger numbers to ensure they remained contemporary over the duration of the study (∼18 months). PN studies were designed to obtain a power >80% if group sizes were six rats, UNaV in rats receiving atrasentan was 50 ± 25% [26] of the expected value in controls [22], and medullary blood flow was 20 ± 5% greater in controls over atrasentan-treated rats [27]. Additional rats were included in every group to account for an expected dropout rate of 25% due to experimental mortality.

Rats receiving vehicle formed the control group for each set of statistical comparisons. Non-normal data (Anderson-Darling test) were compared following log or square-root transformation to generate a Gaussian-distributed data set amenable to parametric analysis, or, where this was not possible, non-parametric testing.

For acute PN studies, baseline mean BP (MBP) was the independent variable used as a surrogate marker of renal perfusion pressure. After confirming that baseline MBP and increments for all groups were similar to control, data were compared with control with two-way repeated measures analysis of variance (ANOVA). The results are presented as the effect on dependent variables from ligation (all rats), ET receptor antagonist and the interaction between the two of them. Where there was an effect from an ET receptor antagonist, Dunnett’s *post hoc* tests were applied to determine whether this effect was observed during individual clearance periods. As additional analysis, dependent variables were plotted against MBP and compared using regression analysis. Linear regression, including polynomial and reciprocal terms, was used first to model curves, and if an adequate fit could not be obtained, non-linear regression was attempted. Regression lines were compared by analysis of covariance (ANCOVA) with Tukey *post hoc* tests (linear) and extra sum of squares *F*-tests (nonlinear), to determine whether one regression line fitted all data sets.

For radiotelemetry, BP and heart rate were compared with the values obtained during the previous recording period, using one-way repeated measures ANOVA, with rat identification as a random factor, and Sidak’s *post hoc* tests.

For all ANOVAs, data were transformed where necessary to generate normality and equality of variance in residuals. For all tests, statistical significance was set at *P*<0.05.

## Results

### Effects of ET receptor antagonists on sodium excretion

**Figure 1:**
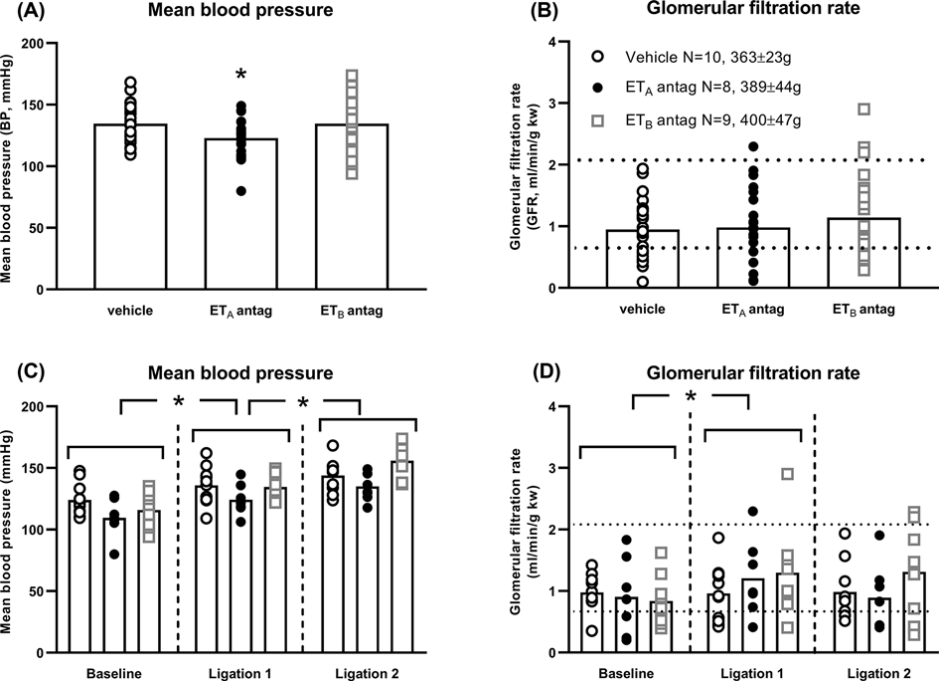
Experimental acute pressure natriuresis (PN) in Sprague Dawley rats after endothelin A (ET_A_) and ET_B_ receptor antagonism. Mean blood pressure (BP) and glomerular filtration rate (GFR) (**A**) Mean BP, group effects. ET blockade modified mean BP (*P*<0.001). The ET_A_ antagonist reduced mean BP by around 12 mmHg compared with vehicle (*P*=0.005). (**B**) GFR, group effects. ET antagonists did not modify GFR (*P*=0.548). Most values remained within an autoregulatory range (horizontal dotted lines) previously described for Sprague Dawley rats during experimental PN [22]. Weights are shown. There was no difference in weight between groups (*P*=0.122). (**C**) Mean BP during individual clearance periods. Arterial ligation increased mean BP (*P*<0.001) above the mean BP of the previous clearance period (ligation 1, *P*<0.001; ligation 2, *P*=0.003). Mean BP and ramps in mean BP were similar between groups during every clearance period (interaction *P*=0.347). (**D**) GFR during individual clearance periods. Arterial ligation increased GFR (*P*=0.004) after ligation 1 (*P*=0.007) but not ligation 2 (*P*=0.977). GFR did not differ between groups during every clearance period (interaction *P*=0.127). Most values remained within an autoregulatory range (horizontal dotted lines) previously described for Sprague Dawley rats during experimental PN [22]. Every rat contributed three data points into every panel (baseline and after ligations 1 and 2). In panels (A and B), they are combined. In panels (C and D), there is one data point per rat per time-point. Bars show mean; vertical dashed lines divide clearance periods; *P<0.05 compared with vehicle or previous clearance period. All comparisons were made with two-way repeated measures analysis of variance (ANOVA) with Dunnett’s *post hoc* tests.

**Figure 2:**
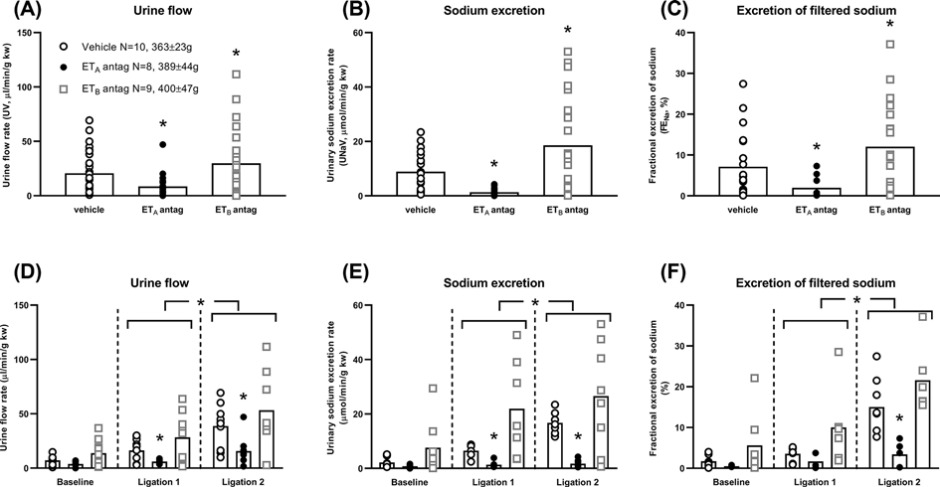
Experimental acute pressure natriuresis (PN) in Sprague Dawley rats after endothelin A (ET_A_) and ET_B_ receptor antagonism (**A**) Pressure diuresis, group effects. ET antagonists modified urine flow rate (UV, *P*<0.001). The ET_A_ antagonist reduced UV (*P*=0.016) and the ET_B_ antagonist increased UV (*P*=0.027). Weights are shown. (**B**) PN, group effects. ET antagonists modified urinary sodium excretion rate (UNaV, *P*<0.001). The ET_A_ antagonist reduced UNaV (*P*=0.035) and the ET_B_ antagonist increased UNaV (*P*=0.001). (**C**) Fractional excretion of sodium (FENa), group effects. ET antagonists modified FENa (*P*<0.001). The ET_A_ antagonist reduced FENa (*P*=0.039) and the ET_B_ antagonist increased FENa (*P*=0.006). (**D**) Pressure diuresis during individual clearance periods. Arterial ligation increased UV (*P*<0.001), increasing the UV after ligation 2 above the UV after ligation 1 (*P*<0.001). UV was reduced by the ET_A_ receptor antagonist after ligation 1 (*P*=0.031) and ligation 2 (*P*=0.029). The increase in UV in ET_B_ receptor antagonist-treated rats after each ligation did not reach significance. (**E**) PN during individual clearance periods. Arterial ligation increased UNaV (*P*=0.002), increasing the UNaV after ligation 2 above the UNaV after ligation 1 (*P*=0.021). UNaV was reduced by the ET_A_ receptor antagonist after ligation 1 and ligation 2 (both *P*<0.001). The increase in UNaV in ET_B_ receptor antagonist-treated rats after each ligation did not reach significance. (**F**) FENa during individual clearance periods. Arterial ligation increased FENa (*P*<0.001), increasing the FENa after ligation 2 above the FENa after ligation 1 (*P*=0.039). FENa was reduced by the ET_A_ receptor antagonist after ligation 2 (*P*=0.005). The increase in FENa in ET_B_ receptor antagonist-treated rats after each ligation did not reach significance. Every rat contributed three data points into every panel (baseline and after ligations 1 and 2). In panels (A, B and C), they are combined. In panels (D, E and F), there is one data point per rat per time-point. Bars show mean; vertical dashed lines divide clearance periods; *P<0.05 compared with vehicle, the previous clearance period or compared with vehicle during the same clearance period. All comparisons were made with two-way repeated measures analysis of variance (ANOVA) with Dunnett’s *post hoc* tests.

PN was induced acutely in all groups of rats when MBP was increased (*P*<0.001; Figure 1A,C, Supplementary Figure S1A, S1C). Ramps of 15 ± 10 mmHg (ligation 1) and a further 11 ± 9 mmHg (ligation 2; both *P*<0.001; Figure 1C, Supplementary Figure S1C) increased UV (*P*<0.001), UNaV (*P*=0.002) and FENa (*P*<0.001) but the effect sizes varied according to ET receptor blockade (Figure 2 and Supplementary Figure S2).

**Figure 3:**
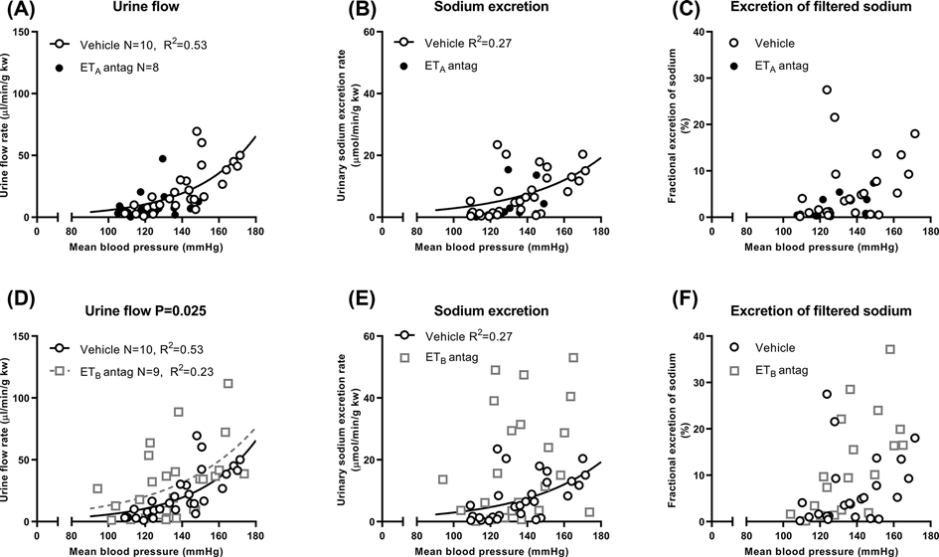
Regression analysis of responses following induction of experimental pressure natriuresis (PN) in Sprague Dawley rats after endothelin A (ET_A_) and ET_B_ receptor antagonism (**A**) Pressure diuresis responses. A curve could not be fitted to the dataset from ET_A_ receptor antagonist-treated rats, which lay mainly below the vehicle-treated pressure diuresis curve. (**B**) PN responses. A curve could not be fitted to the dataset from ET_A_ receptor antagonist-treated rats, which lay mainly below the vehicle-treated PN curve. (**C**) A curve for fractional excretion of sodium (FENa) could not be fitted to the dataset from either ET_A_ receptor antagonist- or vehicle-treated rats. The largest values of FENa occurred following treatment with vehicle. (**D**) Pressure diuresis responses. The pressure diuresis curve was shifted upwards following treatment with the ET_B_ receptor antagonist, compared with vehicle. (**E**) PN responses. A curve could not be fitted to the dataset from ET_B_ receptor antagonist-treated rats, which lay mainly above the vehicle-treated PN curve. (**F**) A curve for fractional excretion of sodium (FENa) could not be fitted to the datasets from either ET_B_ receptor antagonist- or vehicle-treated rats. The largest values of FENa occurred following treatment with the ET_B_ receptor antagonist. Linear (*R*^2^) or non-linear regression (standard deviation of the residuals) was only performed where an adequate goodness-of-fit could be obtained. When this was possible for vehicle-treated and ET receptor antagonist-treated data sets, both curves were compared by analysis of covariance (ANCOVA) with extra sum of squares *F*-tests. The *P* value shown in panel (D) demonstrates that a different curve was required for each data set (*P*<0.05). Every rat contributed three data points into every panel (baseline and after ligations 1 and 2).

Atrasentan markedly suppressed PN. UV, UNaV and FENa (Figure 2A–C) were all reduced by between 60 and 90% (UV, *P*=0.016; UNaV, *P*=0.035; FENa, *P*=0.039). This was apparent for UV (*P*=0.031 and 0.029) and UNaV (both *P*<0.001) after each arterial ligation and for FENa (*P*=0.003) after the second ligation (Figure 2D–F). Curvilinear relationships of UV and UNaV with MBP were all lost (Figure 3A,B).

By contrast, A-192621 enhanced PN. UV, UNaV and FENa (Figure 2A–C) were all increased by 25–40% (UV, *P*=0.027; UNaV, *P*=0.001; FENa, *P*=0.006). These effects did not reach significance during individual clearance periods (Figure 2D–F), but there was an upward shift in the pressure diuresis curve, and values for UNaV and FENa at similar MBPs were increased (Figure 3D–F).

Combined ET receptor blockade with atrasentan and A-192621 gave a similar pattern of results to atrasentan alone. Natriuresis was blunted by ∼70–90% compared with vehicle after the second ligation (UNaV *P*=0.012, FENa *P*=0.002; Supplementary Figure S2B, C, E, F).

### Effects of ET receptor antagonists on renal haemodynamics

MBP (interaction *P*=0.347) and GFR (interaction *P*=0.127) were similar across all groups during individual clearance periods (Figure 1C,D). Ramps in MBP increased medullary flux (*P*=0.042) but did not affect cortical flux (*P*=0.379) or renal artery flow (*P*=0.701).

**Figure 4:**
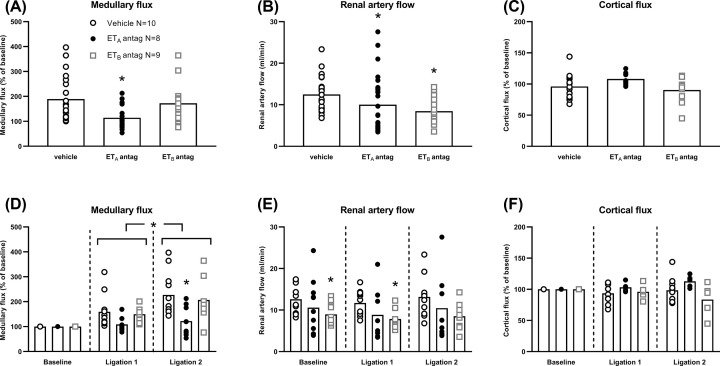
Renal blood flow during experimental pressure natriuresis (PN) in Sprague Dawley rats after endothelin A (ET_A_) and ET_B_ receptor antagonism (**A**) Medullary flux, group effects. ET antagonists modified medullary flux (*P*<0.001). The ET_A_ antagonist reduced medullary flux (*P*=0.001) but the ET_B_ antagonist had no effect (*P*=0.819). (**B**) Renal artery flow, group effects. ET antagonists modified renal artery flow (*P*<0.001). The ET_A_ and the ET_B_ antagonists both reduced renal artery flow (ET_A_*P*=0.014, ET_B_*P*=0.003). (**C**) Cortical flux, group effects. Neither ET antagonist modified cortical flux (ET_A_*P*=0.378, ET_B_*P*=0.818). (**D**) Medullary flux during individual clearance periods. Arterial ligation increased medullary flux (*P*=0.042), increasing the medullary flux after ligation 2 above the medullary flux after ligation 1 (*P*=0.042). Medullary flux was reduced by the ET_A_ receptor antagonist after ligation 2 (*P*=0.002). (**E**) Renal artery flow during individual clearance periods. Arterial ligation did not modify renal artery flow (*P*=0.701). Renal artery flow was reduced by the ET_B_ receptor antagonist after ligation 1 (*P*=0.012) and ligation 2 (*P*=0.029). (**F**) Cortical flux during individual clearance periods. Arterial ligation did not modify cortical flux (*P*=0.379). Cortical flux was not modified by ET antagonists after ligation 1 or ligation 2. Every rat contributed two (panels A, C, D and F) or three data points (panels B and E) into every panel (baseline and after ligations 1 and 2). In panels A, B and C, they are combined. In panels D, E and F, there is one data point per rat per time-point. Bars show mean; vertical dashed lines divide clearance periods; *P<0.05 compared with the previous clearance period or compared with vehicle during the same clearance period. All comparisons were made with two-way repeated measures analysis of variance (ANOVA) with Dunnett’s *post hoc* tests.

**Figure 5:**
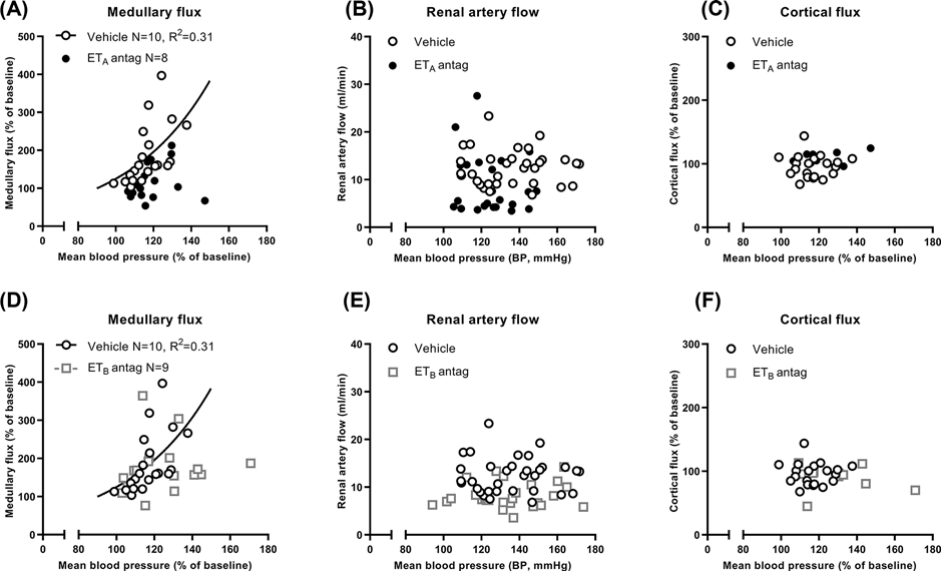
Regression analysis of renal perfusion responses following induction of experimental pressure natriuresis (PN) in Sprague Dawley rats after endothelin A (ET_A_) and ET_B_, receptor antagonism (**A**) Medullary flux. A curve could not be fitted to the dataset from ET_A_ receptor antagonist-treated rats, which lay below the vehicle-treated medullary flux curve. (**B**) Renal artery flow. A curve for renal artery flow could not be fitted to the dataset from either ET_A_ receptor antagonist- or vehicle-treated rats. (**C**) Cortical flux. A curve for cortical flux could not be fitted to the dataset from either ET_A_ receptor antagonist- or vehicle-treated rats. (**D**) Medullary flux. A curve could not be fitted to the dataset from ET_B_ receptor antagonist-treated rats, which lay above and below the vehicle-treated medullary flux curve. (**E**) Renal artery flow. A curve could not be fitted to the datasets from either ET_B_ receptor antagonist- or vehicle-treated rats. (**F**) Cortical flux. A curve could not be fitted to the datasets from either ET_B_ receptor antagonist- or vehicle-treated rats. Linear (*R*^2^) or non-linear regression (standard deviation of the residuals) was only performed where an adequate goodness-of-fit could be obtained. Every rat contributed two (panels A, C, D and F) or three data points (panels B and E) into every panel (baseline and after ligations 1 and 2).

Atrasentan suppressed medullary flux during PN (*P*=0.001, Figure 4A). It increased by only ∼20% from baseline, reaching only half of vehicle levels after ligation 2 (*P*=0.002; Figure 4D). The regression curve of proportionate increases in medullary flux and MBP that was obtained with vehicle was lost with atrasentan (Figure 5A). Renal artery flow was decreased (*P*=0.014; Figure 4B) but was no different to vehicle during individual clearance periods (Figure 4E). Cortical flux was unaffected (*P*=0.378; Figures 4C,F and 5C).

A-192621, however, did not suppress the two-fold increase in medullary flux observed with vehicle (*P*=0.819; Figure 4A) although the curvilinear relationship of medullary flux with MBP was lost (Figure 5D). Renal artery flow was decreased (*P*=0.003; Figure 4B) by around one third at baseline (*P*=0.012) and after ligation 1 by ∼4 ml/min, *P*=0.029; Figure 4E), but there was no influence on cortical flux, which remained close to baseline levels (Figure 4C,F).

With combined ET receptor blockade, suppression of the increase in medullary flux was, again, similar to atrasentan (*P*=0.002; Supplementary Figure S4A,D). However, unlike all the other groups, cortical flux increased overall to ∼25% greater than vehicle (*P*=0.001; Supplementary Figure S4C). This was most marked after the second ligation (*P*=0.003; Supplementary Figure S4F).

### Effects of salt and ET receptor antagonists on BP

**Figure 6:**
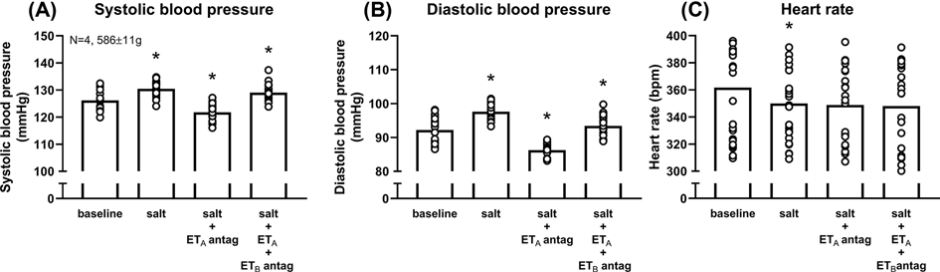
Systolic (SBP) and diastolic (DBP) blood pressures and heart rate in Sprague Dawley rats at baseline and during supplementation with salt, salt + endothelin A (ET_A_) receptor antagonist, and salt + ET_A_ receptor antagonist + ET_B_ receptor antagonist (**A**) Salt and ET receptor antagonists modified SBP (*P*<0.001). Dietary salt supplementation increased SBP, additional ET_A_ receptor blockade decreased SBP, and additional ET_B_ receptor blockade increased SBP (all *P*<0.001). Weights at the end of the study are shown. (**B**) Salt and ET receptor antagonists modified DBP (*P*<0.001). Dietary salt supplementation increased DBP, additional ET_A_ receptor blockade decreased DBP, and additional ET_B_ receptor blockade increased DBP (all *P*<0.001). (**C**) Salt and ET receptor antagonists modified heart rate (*P*<0.001). Dietary salt supplementation decreased heart rate (*P*=0.003), but it was unaffected by additional ET_A_ and ET_B_ receptor blockade (ET_A_*P*=0.500; ET_B_*P*=0.986). Every data point is a single hourly BP or heart rate measurement in one rat over five days. Every rat contributed 120 data points. The horizontal line is the mean. Comparisons between recording periods were made with one-way repeated measures analysis of variance (ANOVA) with Sidak’s *post hoc* tests; *P<0.05 compared with previous recording period.

Salt increased SBP and DBP by ∼3–5 mmHg, and heart rate decreased by ∼15 beats/min (Figure 6A–C).

Addition of atrasentan, reduced average SBP and DBP by ∼8–11 mmHg (both *P*<0.001; Figure 6A,B), but heart rate was unaffected (Figure 6C).

Addition of A-192621 reversed the effects of atrasentan. Average SBP and DBP increased by ∼7–8 mmHg (both *P*<0.001; Figure 6A,B), again, without affecting heart rate (Figure 6C).

## Discussion

The major finding of our study was that administration of the ET_A_ receptor antagonist, atrasentan, severely suppressed PN but nevertheless reversed the salt-induced increase in systolic and diastolic BP. In clinical trials, selective ET_A_ receptor antagonists also lower BP but consistently cause sodium and water retention [19]. Our study provides mechanistic insight, indicating that fluid retention may reflect attenuated PN due to vascular ET_A_ blockade rather than inappropriate sodium reabsorption from off-target blockade of ET_B_ receptors in the distal renal tubule.

The acute PN relationship can be determined experimentally by sequentially increasing BP (and thus renal perfusion pressure), which substantially increases blood flow through the renal medulla and induces a rapid, marked natriuresis [21]. Intact glomerular autoregulation limits the rise in GFR and filtered sodium load [27], so natriuresis mostly reflects decreased renal tubular sodium transport in response to augmented intrarenal hydrostatic pressure and pressure-mediated release of autocrine and paracrine agents. Therefore, suppression of PN can occur if there is a decrease in medullary blood flow or the signaling that it activates, or through a direct effect on sodium transporter activity itself. Here, we demonstrated that suppression of PN by atrasentan was linked with suppression of the normal increase in medullary blood flow.

We considered that this might reflect a systemic vasodilatatory response to atrasentan that reduced the hemodynamic load on the kidneys since, overall, MBP and renal artery flow were decreased in the atrasentan group. However, during individual clearance periods, we did not find that atrasentan reduced renal artery flow, and the small number of GFR data points that were above and below the autoregulatory range were evenly distributed across the vehicle and ET receptor antagonist-treated groups. This suggests that the reduction in circulatory capacity following ligation of major arteries overwhelmed any vasodilatatory response to atrasentan. In addition, atrasentan suppressed diuretic and natriuretic responses at BPs, renal artery flow rates and GFRs similar to those in vehicle-treated rats, and, when it was combined with A-192621 to block both ET_A_ and ET_B_ receptors, medullary blood flow and natriuresis were suppressed without any reduction in renal artery flow.

We also considered that vasodilatation with atrasentan might promote sodium and water retention through increased renal sympathetic activity, activation of the renin–angiotensin–aldosterone system (RAAS), or release of vasopressin [28]. Although we did not adopt alpha blockade because of potential confounding systemic effects on BP and RAAS that occur even when administered intrarenally [29,30], renal sympathetic activity should have been minimal following stripping of the renal artery. Hormonal clamping was not employed because circulatory levels of hormones above physiological ranges [21] could have additional confounding effects, and because it is difficult to determine which hormones should be included and which should not [28]. We have previously shown that our protocol for inducing PN does not modify urinary aldosterone excretion [22], a marker of RAAS activity, and angiotensin II-mediated sodium and water retention would be expected to decrease rather than increase following ET_A_ receptor blockade [10]. Plasma vasopressin levels were not measured. Vasopressin is released during ET_A_ blockade following overstimulation of unblocked endothelial ET_B_ receptors [28]. However, similar suppression of medullary blood flow and natriuresis were observed during combined ET receptor blockade. Therefore, we do not believe that the small overall reduction in renal artery flow, increased renal sympathetic activity, RAAS activation or vasopressin release explain the magnitude of the suppression of PN observed with atrasentan. Instead, we conclude that atrasentan decreased sensitivity to the BP signal, and decreased natriuresis by interfering with ET_A_-receptor mediated regulation of intra-renal blood flow, and, subsequently, decreased tubular sodium transport.

By contrast, and somewhat surprisingly, blockade of ET_B_ receptors with A-192621, enhanced the natriuretic response, despite a slight decrease in renal artery flow, and no influence on the normal increase in renal medullary blood flow that initiates natriuresis. This not only suggested that A-192621 was augmenting the inhibitory influence that increased medullary blood flow exerts on tubular sodium transport but also that the suppression of PN by atrasentan was not due to reduced renal artery flow or off-target ET_B_ receptor blockade. We conclude that ET_B_ receptors inhibit natriuresis when BP is acutely increased.

Pro-natriuretic ET_A_ receptors and anti-natriuretic ET_B_ receptors are at odds with their relative putative roles [10] and clinical applications of ET receptor blockade. Potent, long-lasting ET_A_ receptor-mediated vasoconstriction and inhibition of natriuresis [31] is generally regarded as deleterious in the context of BP regulation [32], while renal tubular ET_B_ receptors promote natriuresis at lower BPs than those reached during experimental PN, possibly through a combination of ENaC inhibition and renal medullary vasodilatation that is enhanced by high salt intake [12,33]. However, two previous studies have also demonstrated anti-natriuretic responses to ET_A_ receptor blockade and pro-natriuretic effects during ET_B_ receptor blockade, although mechanisms were not identified. In those studies, rats were sodium-loaded by i.v. [26] or intra-renal [24] routes, but renal blood flow was not measured. Our study proposes physiological mechanisms to explain these discrepancies: namely that if BP or sodium intake is sufficiently increased, stimulation of vascular ET_A_ receptors promotes sodium excretion by increasing medullary perfusion and initiating PN. ET_B_ receptors, however, inhibit the natriuretic response by promoting tubular sodium reabsorption, either by interfering with pro-natriuretic signaling initiated by increased medullary perfusion or by directly promoting sodium transporter activity. There is already evidence that ET_B_ receptors within the proximal tubule increase sodium transport through sodium-potassium ATPase, the sodium-hydrogen anti-porter (NHE3), and the sodium-glucose transporter 2 (SGLT2) [34–36]. However, ET_A_ receptors that mediate constriction of the medullary *vasa recta* by pericytes [37] would reduce rather than increase medullary perfusion. Therefore, we speculate that ET_A_ receptors upstream of the *vasa recta* increase medullary perfusion and are only activated when BP increases acutely. ET-1 is known to constrict cortical and juxtamedullary afferent and efferent arterioles *in vitro* via both ET receptor subtypes [38,39], and intravital microscopy during infusion with ET-1 and the selective ET_A_ receptor antagonist, BQ123, suggests a greater vasoconstrictive response in the afferent arterioles supplying cortical glomeruli [40]. This could promote diversion of flow through juxtamedullary glomeruli and into medullary *vasa recta* during ET_A_ receptor activation. Support for such a mechanism comes from the sodium and water retention observed in knockout mice that lack vascular smooth muscle ET_A_ receptor signalling [20]. A pro-natriuretic role for the small number of ET_A_ receptors present within the collecting duct has also been proposed [41]. However, our data suggest that loss of ET_A_ receptor-mediated PN can overwhelm the loss of anti-natriuretic effects mediated by renal ET_A_ receptors [31,41,42] and might contribute to the sodium and water retention observed clinically with ET_A_ receptor antagonists [43]. This would explain why this adverse effect is observed even with highly selective ET_A_ antagonists and is unlikely to be eliminated by further increasing ET_A_ receptor selectivity.

Using radiotelemetry, the gold-standard approach to measuring BP in rodents [44], we found that a high salt intake over several days increased BP, confirming a previous study that identified salt-sensitivity in Sprague Dawley rats [45]. We confirmed the BP lowering effect of atrasentan, consistent with vasodilatation, previously described in water and salt-loaded rats [31]. We also confirmed the increase in BP from additional A-192621 that is consistent with the putative vasodilatory and pro-natriuretic roles of ET_B_ receptors. An important outcome of our study is that these effects on BP, recorded over days, were in opposition to those predicted by the Guyton hypothesis and the PN data, recorded over hours. This is directly relevant to the current debate surrounding the relative contributions that PN and vasodilatation make to BP regulation [6]. It suggests that the Guyton hypothesis does not predict the effect of increased dietary salt on BP in the face of salt-sensitivity, and is consistent with recently published data demonstrating an enhanced rather than attenuated PN response in salt-sensitive C57BL/6J mice [46]. Instead, the picture that emerges from our study is that the BP set-point is established by a systemic vascular response that can be manipulated by dietary salt and ET receptor blockade.

The Guyton hypothesis may still have clinical relevance. Pure vasodilator agents, such as dihydropyridine calcium channel antagonists, elicit an increase in heart rate in response to a reduction in BP [47]. In our radiotelemetry study, when atrasentan reduced BP there was no change in heart rate, which would be consistent with an increase in cardiac preload from impaired PN, in opposition to the new BP set-point. Thus, the Guyton hypothesis and PN may provide insight into the systemic hemodynamic load that target organs experience rather than BP, *per se*. Specifically within the context of therapeutic ET_A_ receptor blockade, enhanced vasodilatation may be achieved at the cost of disrupted PN and the inability to efficiently excrete acute sodium and fluid loads. This would explain the apparent paradox of reduced BP but also sodium and water retention observed clinically with ET_A_ receptor antagonists [19].

## Limitations

A limitation of our work is that the experimental studies were performed exclusively in healthy, male rats. Although ET_A_ receptor blockade also reduces sodium excretion in salt-challenged female rats [48], the contribution that ET-1-mediated changes in medullary blood flow make to PN may be less in females than males [49]. In addition, the effect of ET receptor blockade on BP and PN may be different in disease states and following chronic salt and ET antagonist administration. Therefore, the effect of atrasentan on medullary blood flow during experimental PN in female rats, in both sexes in disease states, and after chronic salt-loading and ET receptor blockade should be the subject of further study.

Our conclusions are based on the assumption that *in vitro* receptor affinity and selectivity are maintained *in vivo*. We mitigated for loss of receptor selectivity in the PN and radiotelemetry studies by including combined ET receptor blockade groups.

The potential implications for cardiovascular risk from our data remain to be resolved. The recent SONAR study [50] achieved reductions in cardiovascular risk associated with decreasing proteinuria, suggesting that benefits from blocking ET_A_ receptor-mediated glomerular inflammation and fibrosis in diabetic CKD [51,52] outweigh any deleterious effects on sodium excretion that may occur in the clinical setting. Nevertheless, our study supports the use of ambulatory BP measurement and measurement of sodium intake and excretion to allow detailed analysis of daily fluctuations in BP and sodium balance when determining the impact of pharmacological interventions on cardiovascular risk.

In summary, regulation of BP by ET-1 is complex. It includes opposing and non-putative roles of both ET receptor subtypes at the levels of the renal and non-renal vasculature, and sodium transport. The influence that ET-1 has on BP is dynamic, according to salt intake and the level of BP. We propose that while blockade of ET_A_ receptors may reduce BP, highly selective ET_A_ receptor antagonists may also interfere with renal sodium handling, which might lead to adverse effects of salt and water retention that would offset any beneficial effects on cardiovascular risk.

## Clinical perspectives

- ET_A_ receptor antagonists have considerable clinical potential in resistant hypertension and chronic kidney disease because they reduce BP and proteinuria but patients also develop sodium and water retention by an unknown mechanism.
- Although ET_A_ blockade decreased BP in salt-fed rats, and ET_B_ blockade reversed these effects, novel and opposing effects of ET_A_ (pro-natriuretic) and ET_B_ (anti-natriuretic) receptors were identified during experimental PN.
- Suppression of PN may explain the sodium and water retention observed clinically with ET_A_ receptor antagonists, despite reductions in BP. Increasing ET_A_ receptor selectivity of antagonists may further decrease BP but would not eliminate sodium and water retention.

## Supplementary Material

Supplementary Figures S1-S5

## Data Availability

The data that support the findings of this study are available from the corresponding author upon reasonable request.

## Open Access

Open access for this article was enabled by the participation of The University of Edinburgh in an all-inclusive *Read & Publish* pilot with Portland Press and the Biochemical Society under a transformative agreement with JISC.

## Abbreviations

ANCOVA: analysis of covariance
ANOVA: analysis of variance
BP: blood pressure
DBP: diastolic blood pressure
ET: endothelin
FENa: fractional excretion of sodium
FITC: fluorescein isothiocyanate
GFR: glomerular filtration rate
MBP: mean blood pressure
NHE3: sodium-hydrogen antiporter
PN: pressure natriuresis
SBP: systolic blood pressure
SGLT2: sodium glucose transporter 2
UNaV: urinary sodium excretion rate
UV: urine flow rate
