# Supplementary material for: The acute pressure natriuresis response is suppressed by selective ETA receptor blockade
Source: Clin Sci (Lond). 2022 Jan 5;136(1):15–28. doi: 10.1042/CS20210937 (PMC8734438; doi:10.1042/CS20210937)
Supplement: Supplementary Figures S1-S5 [file CS-2021-0937_supp.pdf]

The acute pressure natriuresis response is suppressed by selective ET_A receptor blockade

Geoffrey J. Culshaw, David Binnie, Neeraj Dhaun, Patrick W.F. Hadoke, Matthew A. Bailey, David J. Webb

University of Edinburgh/British Heart Foundation Centre for Cardiovascular Science, The Queen's Medical Research Institute, The University of Edinburgh, 47 Little France Crescent, Edinburgh, United Kingdom, EH16 4TJ

Short title: Pressure natriuresis, BP and ET-1 blockade

Word count: 1,045

Number of figures: 5

Author for correspondence:

Geoffrey J. Culshaw

Geoff.Culshaw@ed.ac.uk

Tel: +44 (0)131 242 9100

Fax: +44 (0)131 242 9101

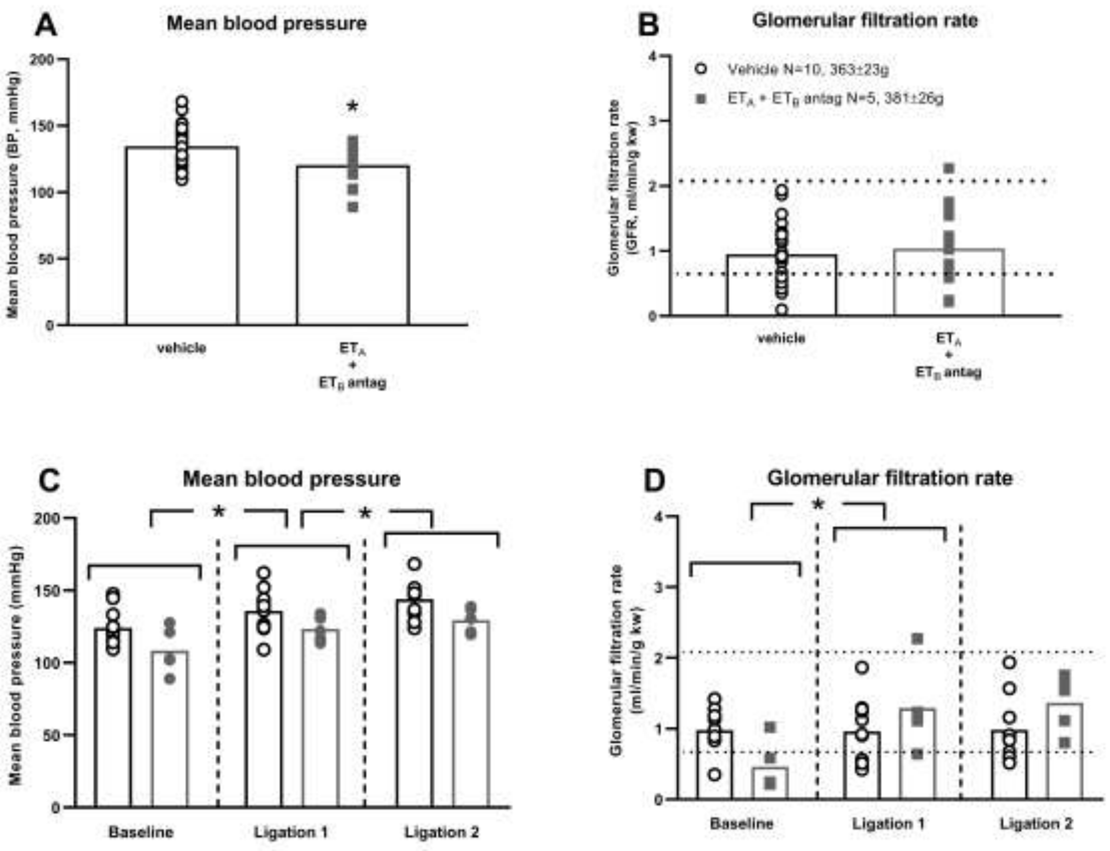
A
Mean blood pressure
Mean blood pressure (BP, mmHg)
200
150
100
50
0
*
vehicle
ET_A + ET_B antag
B
Glomerular filtration rate
Vehicle N=10, 363±23g
ET_A + ET_B antag N=5, 381±26g
Glomerular filtration rate (GFR, ml/min/g kw)
4
3
2
1
0
vehicle
ET_A + ET_B antag
C
Mean blood pressure
Mean blood pressure (mmHg)
200
150
100
50
0
*
*
Baseline
Ligation 1
Ligation 2
D
Glomerular filtration rate
Glomerular filtration rate (ml/min/g kw)
4
3
2
1
0
*
Baseline
Ligation 1
Ligation 2

Supplementary Figure S1. Experimental acute pressure natriuresis (PN) in Sprague Dawley rats after combined endothelin A (ET_A) and ET_B receptor antagonism. Mean blood pressure (BP) and glomerular filtration rate (GFR).

A) Mean BP, group effects. Combined ET blockade reduced mean BP by around 14mmHg compared to vehicle (P=0.005).

B) GFR, group effects. Combined ET blockade did not modify GFR. Most values remained within an autoregulatory range (horizontal dotted lines) previously described for Sprague Dawley rats during experimental PN [22]. Weights are shown. There was no difference in weight compared to vehicle.

C) Mean BP during individual clearance periods. Mean BP and ramps in mean BP were similar between groups during every clearance period.

D) GFR during individual clearance periods. GFR did not differ between groups during every clearance period. Most values remained within an autoregulatory range (horizontal dotted lines) previously described for Sprague Dawley rats during experimental PN [22].

Every rat contributed three data points into every panel (baseline and after ligations 1 and 2). In panels A and B, they are combined. In panels C and D, there is one data point per rat per time-point. Bars show mean; vertical dashed lines divide clearance periods; * = P<0.05 compared with vehicle or previous clearance period. All comparisons were made with two-way repeated measures analysis of variance (ANOVA) with Dunnett's *post hoc* tests.

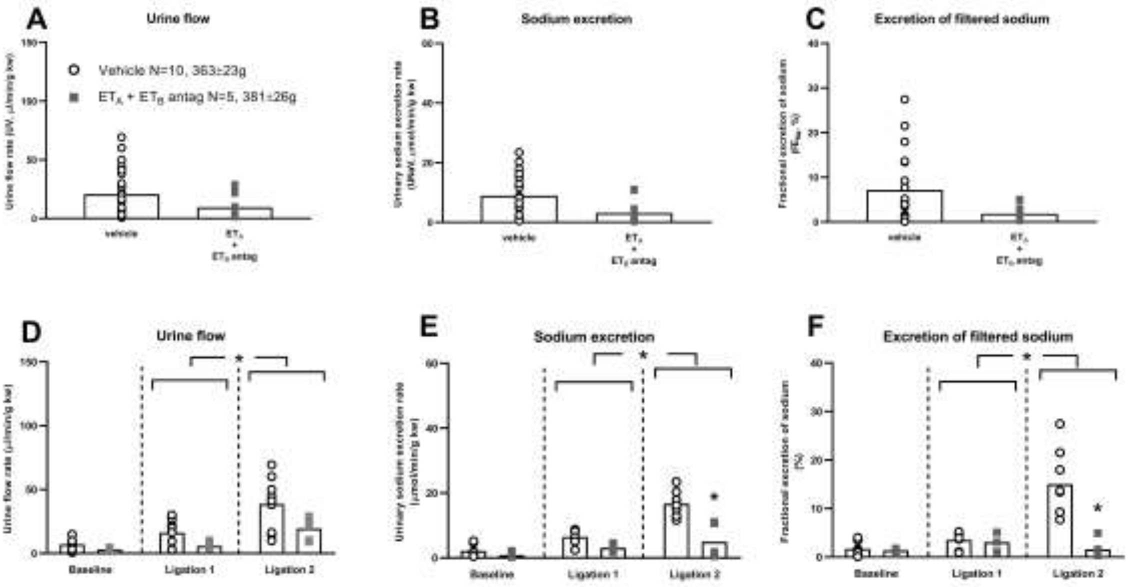
A
Urine flow
Vehicle N=10, 363±23g
ETA + ETB antag N=5, 381±26g
vehicle
B
Sodium excretion
C
Excretion of filtered sodium
D
Urine flow
Baseline
Ligation 1
Ligation 2
E
Sodium excretion
F
Excretion of filtered sodium

Supplementary Figure S2. Experimental acute pressure natriuresis (PN) in Sprague Dawley rats after combined endothelin A (ET_A) and ET_B receptor antagonism.

A) Pressure diuresis, group effects. Reductions in urine flow rate (UV) from combined ET blockade did not reach significance (P=0.071). Weights are shown.

B) PN, group effects. Reductions in urinary sodium excretion rate (UNaV) from combined ET blockade did not reach significance (P=0.245).

C) Fractional excretion of sodium (FENa), group effects. Reductions in FENa from combined ET blockade did not reach significance (P=0.111).

D) Pressure diuresis during individual clearance periods. The reduction in UV from combined ET receptor blockade did not reach significance after either ligation.

E) PN during individual clearance periods. UNaV was reduced by combined ET receptor blockade antagonist after ligation 2 (P=0.012).

F) FENa during individual clearance periods. FENa was reduced by combined ET receptor blockade after ligation 2 (P=0.002).

Every rat contributed three data points into every panel (baseline and after ligations 1 and 2). In panels A, B and C, they are combined. In panels D, E and F, there is one data point per rat per time-point. Bars show mean; vertical dashed lines divide clearance periods; * = P<0.05 compared with the previous clearance period or vehicle during the same clearance period. All comparisons were made with two-way repeated measures analysis of variance (ANOVA) with Dunnett's *post hoc* tests.

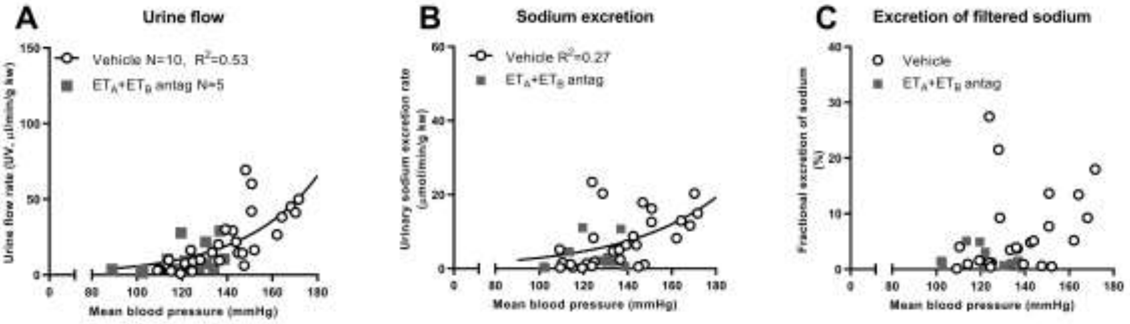
A
Urine flow
Urine flow rate (UV, µl/min/g kw)
Vehicle N=10, R^2=0.53
ET$_A$+ET$_B$ antag N=5
Mean blood pressure (mmHg)
B
Sodium excretion
Urinary sodium excretion rate (µmol/min/g kw)
Vehicle R^2=0.27
ET$_A$+ET$_B$ antag
Mean blood pressure (mmHg)
C
Excretion of filtered sodium
Fractional excretion of sodium (%)
Vehicle
ET$_A$+ET$_B$ antag
Mean blood pressure (mmHg)

Supplementary Figure S3. Regression analysis of responses following induction of experimental pressure natriuresis (PN) in Sprague Dawley rats after combined endothelin A (ET_A) and ET_B receptor antagonism.

A) Pressure diuresis responses. A curve could not be fitted to the dataset from combined ET receptor antagonist-treated rats, which lay mainly below the vehicle-treated pressure diuresis curve.

B) PN responses. A curve could not be fitted to the dataset from combined ET receptor antagonist-treated rats, which lay mainly below the vehicle-treated PN curve.

C) A curve for fractional excretion of sodium (FENa) could not be fitted to the dataset from either combined ET receptor antagonist-treated rats. The largest values of FENa occurred following treatment with vehicle.

D) Pressure diuresis responses. The pressure diuresis curve was shifted upwards following treatment with the ET_B receptor antagonist, compared with vehicle.

Linear (R^2) or non-linear regression (standard deviation of the residuals) was only performed where an adequate goodness-of-fit could be obtained. This was only possible for two vehicle-treated data sets.

Every rat contributed three data points into every panel (baseline and after ligations 1 and 2).

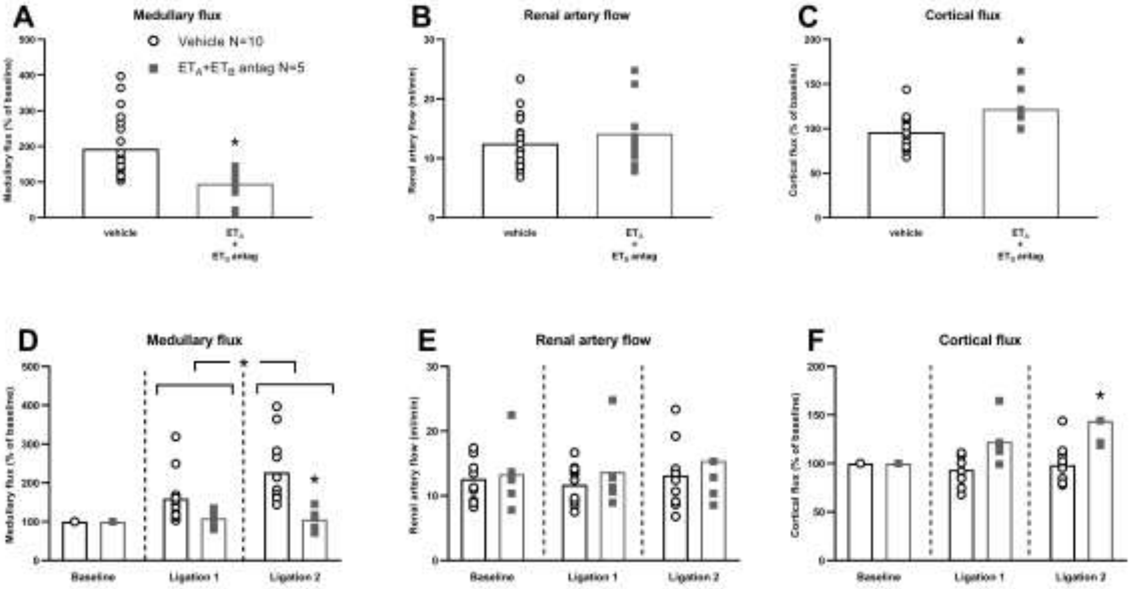
A
Medullary flux
Vehicle N=10
ET_A+ET_B antag N=5
Medullary flux (% of baseline)
vehicle
ET_A + ET_B antag
B
Renal artery flow
Renal artery flow (ml/min)
vehicle
ET_A + ET_B antag
C
Cortical flux
Cortical flux (% of baseline)
vehicle
ET_A + ET_B antag
D
Medullary flux
Medullary flux (% of baseline)
Baseline
Ligation 1
Ligation 2
E
Renal artery flow
Renal artery flow (ml/min)
Baseline
Ligation 1
Ligation 2
F
Cortical flux
Cortical flux (% of baseline)
Baseline
Ligation 1
Ligation 2

Supplementary Figure S4. Renal blood flow during experimental pressure natriuresis (PN) in Sprague Dawley rats after combined endothelin A (ET_A) and ET_B receptor antagonism.

A) Medullary flux, group effects. Combined ET blockade reduced medullary flux (P=0.002).

B) Renal artery flow, group effects. Combined ET blockade did not modify renal artery flow (P=0.769).

C) Cortical flux, group effects. Cortical flux was modified by ET blockade (P<0.001). Combined ET blockade increased cortical flux (P=0.001).

D) Medullary flux during individual clearance periods. Medullary flux was reduced by combined ET receptor blockade after ligation 2 (P=0.002).

E) Renal artery flow during individual clearance periods. Renal artery flow was not modified by combined ET blockade during after either ligation.

F) Cortical flux during individual clearance periods. Cortical flux was increased by combined ET blockade after ligation 2 (P=0.001).

Every rat contributed two (panels A, C, D and F) or three (panels B and E) data points into every panel (baseline and after ligations 1 and 2). In panels A, B and C, they are combined. In panels D, E and F, there is one data point per rat per time-point. Bars show mean; vertical dashed lines divide clearance periods; * = P<0.05 compared with the previous clearance period, vehicle or compared with vehicle during the same clearance period. All comparisons were made with two-way repeated measures analysis of variance (ANOVA) with Dunnett's *post hoc* tests.

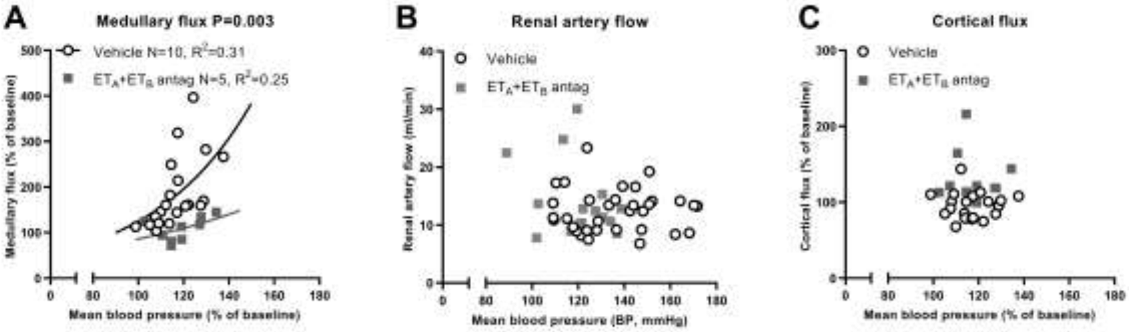

A
Medullary flux P=0.003
Vehicle N=10, R2=0.31
ETA+ETB antag N=5, R2=0.25
Medullary flux (% of baseline)
Mean blood pressure (% of baseline)
B
Renal artery flow
Vehicle
ETA+ETB antag
Renal artery flow (ml/min)
Mean blood pressure (BP, mmHg)
C
Cortical flux
Vehicle
ETA+ETB antag
Cortical flux (% of baseline)
Mean blood pressure (% of baseline)

Supplementary Figure S5. Regression analysis of renal perfusion responses following induction of experimental pressure natriuresis (PN) in Sprague Dawley rats after endothelin A (ET_A) and ET_B, receptor antagonism.

A) Medullary flux. The medullary flux curve was shifted upwards following combined ET receptor blockade.

B) Renal artery flow. A curve for renal artery flow could not be fitted to the dataset from either combined ET receptor antagonist-treated rats or vehicle-treated rats.

C) Cortical flux. A curve for cortical flux could not be fitted to the dataset from either combined ET receptor antagonist-treated rats or vehicle-treated rats.

Linear (R^2) or non-linear regression (standard deviation of the residuals) was only performed where an adequate goodness-of-fit could be obtained. When this was possible for vehicle-treated and combined ET receptor antagonist-treated data sets, both curves were compared by analysis of covariance (ANCOVA) with extra sum of squares F-tests. The P value shown in panel A demonstrates that a different curve was required for each data set ($P<0.05$).

Every rat contributed two (panels A and C) or three (panel B) data points into every panel (baseline and after ligations 1 and 2).
